# Association of trimethylamine N-oxide with coronary atherosclerotic burden in patients with non-ST-segment elevation myocardial infarction

**DOI:** 10.1097/MD.0000000000020794

**Published:** 2020-07-02

**Authors:** Khalid Bin Waleed, Yongkang Lu, Qiang Liu, Fanfang Zeng, Hong Tu, Yi Wei, Shuai Xu, Zhiling Zhang, Yang Rongfeng, Ailing Fan, Afrasyab Altaf, Junlei Chang, Lili Wang

**Affiliations:** a Department of Cardiology, Fuwai Hospital Chinese Academy of Medical Sciences, Shenzhen; b Institute of Biomedicine and Biotechnology, Shenzhen Institutes of Advanced Technology, Chinese Academy of Sciences, Shenzhen, China; c Department of Cardiology, Rehman Medical Institute, Peshawar, Pakistan.

**Keywords:** atherosclerotic burden, trimethylamine N-oxide, non-ST-segment elevation myocardial infarction, primary percutaneous coronary intervention

## Abstract

**Background:**

Recently, trimethylamine N-oxide (TMAO) unexplained gut microbe has been proposed as a promising risk factor for atherosclerotic cardiovascular disease (CVD) pathogenesis and adverse events. The relationship of TMAO with coronary atherosclerotic burden has been evaluated in patients with stable coronary artery disease and ST-segment elevation myocardial infarction, but still needs to be explored in newly diagnosed non-ST-segment elevation myocardial infarction (NSTEMI) patients.

**Material and methods:**

A prospective, single-center, SZ-NSTEMI trial (ChiCTR1900022366) is underway to investigate the relationship of TMAO with the severity and prognosis of coronary atherosclerosis in newly diagnosed NSTEMI patients who will undergo coronary angiography with primary percutaneous coronary intervention (pPCI). The primary endpoint of the study will be assessed the association of TMAO with coronary atherosclerotic severity quantify by the number of diseased coronary arteries and SYNTAX score after the coronary angiography. The secondary endpoints will be identified the TMAO as a prognostic biomarker for the short (1 month) and long-term (12 months) major cardiovascular and cerebrovascular events (MACCEs) rate including myocardial infarction, target vessel revascularization, stroke, heart failure, all-cause rehospitalization, and all-cause mortality after the pPCI. The blood samples will be collected from each patient before the procedure to measure the TMAO by isotope dilution high-performance liquid chromatography. In conclusion, SZ-NSTEMI will be the first cohort that will be investigated the association of TMAO with the severity and prognosis of coronary atherosclerotic burden in NSTEMI patients, aiming to identify TMAO as a predictor and a prognostic biomarker.

## 1 Introduction

### 1.1 Background and rationale

Cardiovascular disease (CVD) is associated with a markedly high incidence in the general population and considered the leading cause of death worldwide.^[1–3]^ Non-ST-segment elevation myocardial infarction (NSTEMI) is the most frequent type of CVD and represents the largest group of patients undergoing primary percutaneous coronary intervention (pPCI).^[1–3]^ Though NSTEMI patients may have lower in-hospital mortality than patients with ST-segment elevation myocardial infarction (STEMI), but long-term mortality is two-fold higher than that in patients with STEMI.^[4,5]^ Moreover, sufficient attention has been given to traditional risk factors, such as age, gender, hypertension, dyslipidemia, smoking, and diabetes, and considerable use of modern therapies only decreased 30% of CVD related mortality and morbidity.^[1–3,6]^ The early risk stratification and optimal treatment strategies are crucial for improving the prognosis of NSTEMI patients. Therefore, an increased demand to explore new CVD pathogenesis risk factors that have not been explained yet in clinical settings.

Recently, plasma the trimethylamine N-oxide (TMAO) unexplained gut microbe has emerged as a promising risk factor for CVD pathogenesis and adverse events.^[7–13]^ The trimethyllysine (TML) a precursor of TMAO was also found both alone and in combination with TMAO an independent and reproducible clinical predictor for the incidence of mortality in CVD patients.^[14]^ TMAO was recently shown to be an independent predictor of a high atherosclerotic burden in patients with STEMI and stable coronary artery disease.^[15,16]^ However, the association of TMAO and TML yet to be investigated in NSTEMI patients. Here, we present the study protocol of the SZ-NSTEMI trial, a prospective, single-center, cohort study which aimed to investigate the relationship of plasma TMAO and TML with an atherosclerotic burden and major cardiovascular and cerebrovascular events (MACCEs) including myocardial infarction, target vessel revascularization, stroke, heart failure, all-cause re-hospitalization, and all-cause mortality after CAG with pPCI in NSTEMI patients.

### 1.2 Study objective

The primary objective of the study is to evaluate the relationship between TMAO and TML levels with the extent of an atherosclerotic burden as quantified by the number of diseased coronary vessels and the SYNTAX score in NSTEMI patients.

The secondary objective of the study is to assess TMAO and TML as prognostic biomarkers for the short (1 month) and long-term (12 months) MACCE rate after the pPCI in NSTEMI patients.

## 2 Methods/design

### 2.1 Study design

This clinical prospective, non-randomized SZ-NSTEMI trial (ChiCTR1900022366) is underway to investigate the relationship of plasma TMAO and TML levels with an atherosclerotic burden and MACCE rate in NSTEMI patients. The study protocol was approved by the Ethics Committee of Fuwai Hospital Chinese Academy of Medical Sciences, Shenzhen (Ethics approval number: YN201901) and registered clinical trial (ChiCTR1900022366). All study procedures comply with the Declaration of Helsinki and all participants will give informed consent after screening inclusion and exclusion criteria before enrollment. The duration of the study is two years which include the selection, initial screen, CAG, pPCI, and the follow-up period for the MACCEs after the pPCI.

### 2.2 Study population

The present work will be enrolled 2 groups of individuals including Study group (200 newly diagnosed NSTEMI patients and having at least single or multiple coronary lesions ≥ 70% after CAG to fulfill eligibility for pPCI) and Control group (100 healthy individuals) at Fuwai Hospital. The Study group already enrolled 70 NSTEMI patients and will be continued to complete the enrollment of 200 patients who will undergo CAG with pPCI. The NSTEMI will be diagnosed according to symptoms, electrocardiogram (ECG) changes and myocardial enzyme results as recommended by European Society of cardiology.^[1–3]^ NSTEMI symptoms will be noted as the presence of acute chest pain or chest tightness or dyspnea with duration over 30 minutes but no persistent ST-segment elevation; ECG changes may include ST-segment depression ≥0.05 mV, T-wave inversion ≥0.3 mV or flat T wave, or transient ST-segment elevation ≤0.05 mV or maybe normal ECG; and myocardial enzyme with positive elevated troponin T and I, and/or CK-MB exceeding the upper limit of the normal range.^[1–3]^ The Control group will be included 100 healthy individuals without known cardiovascular disease or/and non-cardiovascular diseases who will only visit for health screen. The Control group purpose will be provided the reference interval values for TMAO and TML levels compared to the Study group with a ratio of 1:2 respectively.

**Figure 1:**
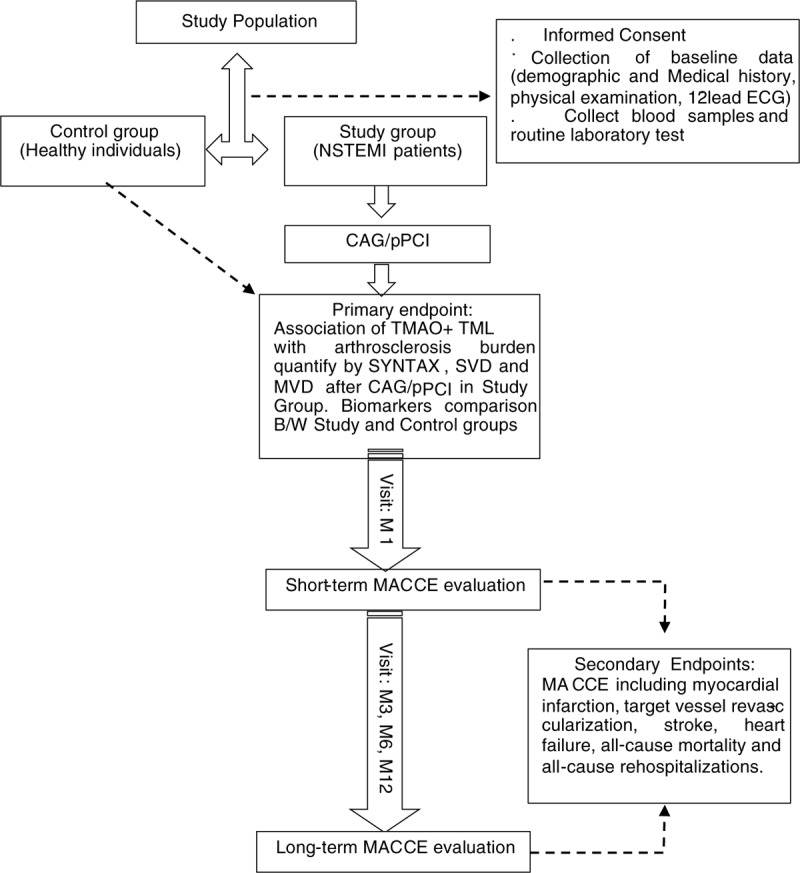
Flow chart diagram of the SZ-NSTEMI study protocol. B/W = between, CAG = coronary angiography, ECG = electrogram, M = month, MACCE = major adverse cardiovascular and cerebrovascular event, MVD = multiple vessels disease, NSTEMI = non ST-segment myocardial infarction, pPCI = primary percutaneous coronary intervention, SVD = single vessel disease, TMAO = trimethylamine N-oxide, TML = trimethyllysine.

The study will be conducted over a period of 2 years in which all included individuals will be examined at baseline and Study Group individuals will be followed up for the short (1 month) and long-term (12 months) MACCE rate after the pPCI, as shown by SZ-NSTEMI diagram with a flowchart that will be used in the present study (Fig. 1).

Inclusion criteria:

1. Newly diagnosed NSTEMI patients without having previous coronary artery disease
2. Having at least single or multiple coronary lesions ≥70% after CAG to fulfill eligibility for pPCI
3. Willing to provide informed consent
4. Both men and women aged between 18 and 75 years old.

Exclusion criteria

1. Patients with a previous history of coronary artery disease including STEMI, NSTEMI, stable or unstable coronary artery disease or percutaneous transluminal coronary angioplasty or PCI or coronary artery bypass surgery
2. Having single coronary lesion < 70% or patient not willing to perform pPCI with single coronary lesion ≥ 70%.
3. Having left main coronary artery lesion
4. Patients present with diagnosis of STEMI, stable or unstable coronary artery disease
5. Patients with severe valvular Heart disease, severe renal and hepatic disease, heart failure, peripheral arterial disease, atrial fibrillation, chronic obstructive lung disease, acute and chronic infection, history of cancer, and history stroke or bleeding within 3 months.
6. Patient unwillingness to participate and not willing to provide informed consent
7. Pregnancy or lactation
8. Conditions related to an estimated life expectancy of less than 1 year.

### 2.3 Study settings

The SZ-NSTEMI study will be conducted at Fuwai Hospital Chinese Academy of Medical Sciences, Shenzhen and is funded by the National Natural Science Foundation of China (No: 81971634) and Shenzhen Basic Science Research Project (No. JCYJ20170413165705083).

### 2.4 Study procedures

The following information will be obtained from all individuals including baseline demographic, present and past medical history, physical examination findings, 12 lead ECG, and cardiovascular risk factors before enrollment.

Diagnostic CAG and pPCI procedures will be performed by the redial approach within 24 hours of admission under the Fuwai Hospital standard protocol. Multiple diagnostic CAG views will be obtained, for the left anterior descending and left circumflex coronary at least four projections, and the right coronary artery at least two projections. All patients will be received standard oral dual antiplatelet therapy with 300 mg of aspirin (follow by 75–100 mg daily) and 600 mg of clopidogrel (follow by 75 mg daily for ≥ 12 months) as well as intravascular infusions of 70 to 100 IU/kg of unfractionated to weight and boost the operation progressed. During pPCI, the choice and selection of balloons, guide wires, type and size of stents will be determined by the operator.

For primary endpoints, 2 experienced interventional cardiologists blinded to study protocol will evaluate the atherosclerotic burden by the SYNTAX score after the CAG. The SYNTAX score will be calculated from the official SYNTAX score website (www.syntaxscore.com/calculator/syntaxscore/frameset.htm) for each NSTEMI patient. All patients will be divided into two groups according to SYNTAX score including low–intermediate risk (SYNTAX score < 22) and high-risk (SYNTAX score ≥22). The opinion of a third interventional cardiologist will be taken if there is any discrepancy between the results of the SYNTAX score. Moreover, a single vessel disease or multiple vessel diseases will be defined as one or more major coronary vessels exhibiting ≥70% stenosis after the procedure.

### 2.5 Blood sampling and biomarkers measurement

Routine and biochemical blood tests will be measured from venous blood samples obtained in a fasting state on the morning following the admission day or before the procedure. An extra EDTA blood sample will also be obtained from each included individual and centrifuged immediately at 2500 g for 10 minutes at room temperature and stored at –80^o^C incubator for later analysis. The plasma TMAO and TML will be measured by isotope dilution high-performance liquid chromatography with online tandem mass spectrometry on an API 3200 triple quadra pole mass spectrometer (AB SCIEX, Framingham, MA) as previously described.^[13–15]^ Laboratory personnel who analyze TMAO, TML and other routine laboratory tests will be blinded to individual characteristics.

### 2.6 Study outcomes

The primary endpoint of the study will be assessed an association of TMAO and TML with coronary atherosclerotic severity quantify by the number of diseased coronary arteries, SYNTAX scores after the CAG. For secondary endpoints in Study group, the plasma TMAO and TML will be used as prognostic biomarkers for MACCEs including myocardial infarction, target vessel revascularization, stroke, heart failure, all-cause re-hospitalization and all-cause mortality at the short (1 month) and long-term (12 months) after the pPCI. Each patient in the Study group will be scheduled for follow-up at 1, 3, 6, and 12 months after pPCI at Fuwai hospital by a trained team of cardiologists to identify MACCEs and further clinical consultation.

### 2.7 Study timeline

The prospective NSTEMI trial is underway from June 2019 and will be completed in June 2021.

1) Baseline (day 0)
  - Obtain consent documents from each individual.
  - Selection of patients according to inclusion and exclusion criteria
  - Obtain demographic information, present, and past medical history, medication history
  - Record results of physical examinations, 12-lead ECG, echocardiography
  - Collect blood samples (complete blood count, biochemistry, and extra EDTA tube for TMAO and TML biomarkers)
  - Undergo CAG and pPCI (Study Group individuals)
2) Short-term (1 month after the pPCI) follow-up visit
  - Record results of physical examinations, 12-lead ECG, transthoracic echocardiology, present medical history, and consultation
  - Short-term MACCE evaluation
3) Long-term (3, 6, and 12 months after the pPCI) visits
  - Record results of physical examinations, 12-lead ECG, transthoracic echocardiology, present medical history, and consultation
  - Long-term MACCE evaluation.

### 2.8 Data collection

All the information will be collected from each participant that consists of patient's background, medical history, medication, blood samples, ECG, echocardiography. The study group will be follow-up for MACCE evaluation after pPCI.

### 2.9 Sample size

The protocol planned to enroll 300 participants including 200 NSTEMI patients in the Study group and 100 healthy individuals in the Control group. The ratio of 2:1 sample size will provide 80% power to detect an absolute difference of TMAO and TML biomarkers between the 2 groups. The 200 NSTEMI patients with given a 10% rate of loss during follow-up for MACCEs as secondary endpoints will provide 80% power at a two-sided significance level of 0.05.

### 2.10 Statistical analysis

Continuous variables will be expressed as mean ± standard deviation or median with interquartile range, whereas categorical data will be expressed as percentages. Continuous variables will be analyzed using *t* tests or Fisher exact or Pearson χ^2^ tests for categorical variables as appropriate. Kolmogorov-Smirnov test will be performed for normality. Pearson and Spearman correlation analyses will be used to examine the correlations between TMAO and TML levels, SYNTAX score and the numbers of diseased coronary vessels. Ordinal logistic regression analyses with adjustments for traditional risk factors (including age, sex, hypertension, diabetes mellitus, smoking, low-density lipoprotein cholesterol level, high-density lipoprotein cholesterol level, and triglyceride and body mass index) will be used to determine the association of TMAO and TML with a high SYNTAX score and the presence of multiple vessel diseases. Kaplan-Meier curves will be used for correlation of TMAO and TML with the short and long-term MACCE rate after the pPCI. A *P* value ≤ .05 will be considered statistically significant. All statistical analyses will be performed with SPSS 25 (IBM).

## 3 Discussion

The use of novel biomarkers has transformed cardiovascular medicine both in diagnosis and risk assessments.^[14,17–20]^ Current risk stratification markers of myocardial necrosis indicate predominantly the short-term adverse risks and recognize those individuals for whom more invasive diagnostic testing and revascularization may be reasonable.^[17–20]^ The traditional CVD risk biomarkers including cholesterol, triglycerides, high-sensitivity C- reactive protein have the ability for long-term risk prediction.^[20]^ There is limited data that could be utilized biomarkers for the prediction of high-risk CVD patients and their prognostic evaluation of the short and long-term outcomes.^[17–20]^ Identification of novel biomarkers that may predict myocardial necrosis as well as provide both short and long-term prognostic values in CVD particularly NSTEMI subjects is alike of interest, as this may enable us to recognize pathways related to CVD pathogenesis, as well as enhanced potential preventive CV risk reduction efforts.^[17–20]^ Here, this proposed SZ-NSTEMI protocol is ongoing which aims to investigate the relationship of TMAO and TML with atherosclerosis burden and prognostic risk ability in NSTEMI patients.

TMAO is the gut microbiota derived from dietary trimethylamines-containing nutrients (mainly choline, phosphatidylcholine, L-carnitine).^[7]^ TMAO promotes macrophages foam cell formation, development of platelet hyperactivity, altered bile acids, and cholesterol transport, and activation of the inflammatory pathway.^[7–13]^ All these factors are linked with an increased risk of many types of cardiovascular diseases including atherosclerosis, stroke, congestive heart failure, atrial fibrillation, and chronic kidney disease.^[7–13]^ TML is a precursor of TMAO was also found alone and together with TMAO with increased risk of short and long-term CVD events in patients with chest pain and STEMI.^[14]^ In animal studies found that TMAO promotes atherosclerosis by inhibiting reverse cholesterol transport, activating macrophages, and increasing platelet activity and thrombosis, whereas inhibiting TMAO production decreases the formation of atherosclerotic lesions.^[8–12]^ Many clinical studies also confirmed the association of TMAO and its precursor TML metabolites with CVD risks after adjusting the traditional risk factors.^[7,13–15]^

More recently, it has been further confirmed that long-term increases in TMAO were related to a greater risk of CVD and repeated assessment of TMAO over 10 years improved the identification of individuals with a higher risk of CVD.^[21]^ A Chinese cohort of 335 STEMI patients found that higher TMAO levels are significantly associated with high atherosclerotic burden assessed by SYNTAX score and multi-vessel disease.^[15]^ Another prospective series of 211 patients with STEMI demonstrated higher TMAO levels association with plaque rupture assessed by optical coherence tomography examination and these findings have significant adverse risks in CVD patients.^[22]^ The association of TMAO with atherosclerosis burden in stable coronary artery disease has also been reported previously.^[21]^ In light of these promising findings, the evaluation TMAO and TML are warranted in NSTEMI patients as represent the most frequent group of CVD patients undergoing pPCI, which may serve as a potential target for atherosclerosis prevention and treatment as compared with present traditional treatments. Here, the SZ-NSTEMI cohort is underway to evaluate TMAO and TML in NSTEMI patients, which may further enable us to stratify low and high-risk patients and may impact the short and long-term treatment protocol at the clinical setting.

As for the limitations, the study will carry out at a single center with a comparable number of NSTEMI patients. Moreover, TMAO and TML are gut microbes that may have the potential to be influenced by nutritional status and diet. However, this study protocol will be unable to evaluate the nutritional status of each participant before enrollment and that may bias the results.

## 4 Conclusions

The SZ-NSTEMI will be the first cohort that will investigate the association of TMAO and TML with the severity and prognosis of coronary atherosclerotic burden in NSTEMI patients, aiming to identify TMAO and TML as predictors and prognostic biomarkers.

## Author contributions

The research design, protocol, submission to ethical committee and trial registration were performed by Lili Wang, Junlei Chang, Khalid Bin Waleed and Yangkong Lu. The patient selection for inclusion and exclusion criteria, informed consent, CAG with pPCI, data collection, and statistical analysis will be performed by Lili Wang, Khalid Bin Waleed, Yongkang Lu, Qiang Liu, Fangfang Zeng, Hong Tu, Yi wei, Shuai Xu, Yang Rongfeng. All authors reviewed the content and approved the final version of the manuscript.

**Conceptualization:** Lili Wang, Junlei Chang, Khalid Bin Waleed, Yongkang Lu, Qiang Liu, Fangfang Zeng, Hong Tu, Yi wei, Shuai Xu, Yang Rongfeng.

**Data curation:** Lili Wang, Junlei Chang, Khalid Bin Waleed, Yongkang Lu, Qiang Liu, Hong Tu.

**Formal analysis:** Lili Wang, Khalid Bin Waleed, Fangfang Zeng, Yi wei, Shuai, Yang Rongfeng, Zhiling Zhang, Ailing Fan, Afrasyab Altaf.

**Funding acquisition:** Lili Wang, Junlei Chang, Khalid Bin Waleed, Yongkang Lu.

**Investigation:** Lili Wang, Junlei Chang, Khalid Bin Waleed, Yongkang Lu, Qiang Liu, Fangfang Zeng, Hong Tu, Yi wei, Shuai Xu, Zhiling Zhang, Yang Rongfeng, Ailing Fan.

**Methodology:** Lili Wang, Chang Junlei, Khalid Bin Waleed, Lu Yongkang, Qiang Liu, Afrasyab Altaf.

**Resources:** Lili Wang, Junlei Chang, Khalid Bin Waleed, Yongkang Lu, Qiang Liu, Fangfang Zeng, Hong Tu.

**Supervision:** Lili Wang, Junlei Chang, Qiang Liu

**Validation:** Lili Wang, Junlei Chang, Qiang Liu, Fangfang Zeng, Hong Tu, Khalid Bin Waleed, Yongkang Lu.

**Visualization:** Lili Wang, Khalid Bin Waleed

**Writing – orginal draft:** Lili Wang, Junlei Chang, Khalid Bin Waleed, Yongkang Lu, Qiang Liu, Fangfang Zeng, Hong Tu, Yi wei, Shuai Xu, Zhiling Zhang, Yang Rongfeng, Ailing Fan, Afrasyab Altaf.
